# Prediction of the onset of mood disorders in the offspring of parents with bipolar and major depressive disorders

**DOI:** 10.1192/j.eurpsy.2026.10335

**Published:** 2026-07-31

**Authors:** M. Preisig, C. Vandeleur

**Affiliations:** 1 Psychiatriy, Lausanne University Hospital; 2 Toises Psychiatric Center, Lausanne, Switzerland

## Abstract

**Objective:**

Although a series of risk factors for mood disorders have been previously identified, research that prospectively and simultaneously tested these factors is still scarce. Using a cohort of offspring of parents with bipolar disorders (BPD) and major depressive disorders (MDD), the goal of the present paper was to simultaneously and prospectively assess the association of sociodemographic characteristics, mood and non-mood disorders in the parents and co-parents, life stressors, personality traits as well as psychopathological precursors with the incidence of (hypo)manic episodes or MDD in offspring.

**Methods:**

We have collected clinical information on 85 probands with BPD, 74 probands with MDD, 22 probands with substance use disorders, 68 controls, 233 co-parents as well as their 451 offspring. The mean age of the offspring at study intake was 10.0 years (s.d: 4.6) and the mean duration of follow-up in offspring was 16.3 (s.d: 6.1) years.

**Results:**

The onset of mania/hypomania in offspring was predicted by the proband’s early onset BPD and oppositional defiant disorder, the occurrence of a major depressive or subthreshold hypomanic episode, whereas the onset of MDD was preceded by sexual abuse or witnessing of trauma to others.

**Conclusion:**

Our data support differential risk factors for (hypo)mania and MDD, further suggesting the specificity of major mood disorders. The strong association between the proband’s early-onset BPD and the incidence of (hypo)mania in offspring suggests the implication of genetic or family-shared risk factors, whereas the development of MDD in offspring is likely to be more attributable to adverse environmental stressors.

**Disclosure of Interest:**

None Declared

